# Effect of Acrylamide Supplementation on the Population of Vasoactive Intestinal Peptide (VIP)-Like Immunoreactive Neurons in the Porcine Small Intestine

**DOI:** 10.3390/ijms21249691

**Published:** 2020-12-18

**Authors:** Katarzyna Palus, Michał Bulc, Jarosław Całka

**Affiliations:** Department of Clinical Physiology, Faculty of Veterinary Medicine, University of Warmia and Mazury in Olsztyn, 10-719 Olsztyn, Poland; michal.bulc@uwm.edu.pl (M.B.); calkaj@uwm.edu.pl (J.C.)

**Keywords:** acrylamide, vasoactive intestinal peptide, small intestine, enteric nervous system, pig

## Abstract

Acrylamide is one of the harmful substances present in food. The present study aimed to establish the effect of acrylamide supplementation in tolerable daily intake (TDI) dose (0.5 µg/kg b.w./day) and a dose ten times higher than TDI (5 µg/kg b.w./day) on the population of vasoactive intestinal peptide-like immunoreactive (VIP-LI) neurons in the porcine small intestine and the degree of the co-localization of VIP with other neuroactive substances (neuronal nitric oxide synthase (nNOS), substance P (SP), and cocaine- and amphetamine-regulated transcript peptide (CART)). In our work, 15 Danish landrace gilts (5 in each experimental group) received capsules (empty or with low or high doses of acrylamide) for a period of 28 days with their morning feeding. Using double immunofluorescence staining, we established that acrylamide supplementation increased the number of neurons showing immunoreactivity towards VIP in all types of enteric nervous system (ENS) plexuses and fragments of the small intestine studied. Moreover, both doses of acrylamide led to changes in the degree of co-localization of VIP with nNOS, SP, and CART in intramural neurons. The observed changes may be the adaptation of neurons to local inflammation, oxidative stress, or the direct toxic effects of acrylamide on intestinal neurons, also referred to as neuronal plasticity.

## 1. Introduction

In recent years, consumer awareness has increased, and stricter legal regulations have forced food producers to control the levels and limit the presence of harmful substances in food that may have a negative impact on human health and life. One of these substances is acrylamide (ACM). The presence of ACM in food was initially detected in 2002 and since then scientists have been trying to develop methods to reduce the compound in food [1]. Large amounts of ACM have been confirmed in high-carbohydrate, heat-treated food products. It is formed in the Maillard reaction between free asparagine and reducing sugars (glucose, fructose) during thermal processing (above 120 °C), such as frying, baking, toasting, grilling, and extrusion [2]. ACM is also formed in the process of roasting coffee beans, cocoa, and cereals [1,2]. It was estimated that people consume from 0.3 to 0.8 µg/kg of body weight of acrylamide contained in food products per day [3]. In the light of previous research, ACM consumed with food causes numerous homeostatic disorders in the body, showing teratogenic, genotoxic, neurotoxic effects, or disorders of reproductive functions [1,4,5]. To date, the results of epidemiological studies have not provided conclusive evidence of the relationship between the dietary intake of ACM and the increased risk of cancer in humans. However, based on available animal studies, the International Agency for Research on Cancer (IARC) classified it as “possibly carcinogenic to humans” (Group 2A) [6]. In turn, there are reports describing the neurotoxic effect of ACM on the human body [7]. Although the gastrointestinal (GI) tract is one of the main ways of ACM absorption [8], many issues related to its effect on the gut remain unclear.

The digestive tract along its entire length possesses intramural neurons grouped into plexuses that represent the enteric nervous system (ENS). It is the third part of the autonomic nervous system and is able to coordinate the work of the GI tract without the involvement of the central nervous system (CNS) [9,10]. The anatomical structure of the plexuses and their arrangement in the GI wall show differences, both interspecies and between digestive tract segments. It has been shown that in large mammals (including pigs), in the upper part of the GI tract (esophagus and stomach), the ENS is organized into two plexuses: the myenteric plexus (MP) and the submucous plexus (SmP). In the small and large intestines, two submucous plexuses are distinguished: the outer submucous plexus (OSP) and the inner submucous plexus (ISP) [10,11].

ENS neurons synthesize and release many neurochemicals that act as neurotransmitters or neuromodulators [9]. Each of neurons, depending on its function, can synthesize more than one neuroactive substance. This phenomenon, called the neurochemical coding of neurons, can distinguish functional classes of neurons, including inhibitory and excitatory motor neurons, vasomotor neurons, interneurons, and sensory neurons [12]. Due to the functional richness and the large number of cells that form the ENS, it is known as the “gut brain” and is able to coordinate many physiological functions in the digestive tract, such as ensuring the optimal secretion of digestive juices through the stomach, local blood flow, intestinal epithelial substance transport, gastrointestinal immune response and inflammatory process, or regulating intestinal peristalsis [10]. Moreover, numerous disorders of the GI tract trigger a response from ENS neurons, which aims at restoring the balance of the body and is expressed mainly as a change in the level of neurotransmitters [11,13,14]. Among the many neuroactive substances described in the ENS structures, one of the most important neuropeptides engaged in the control of both physiological and pathological states in the digestive tract is the vasoactive intestinal peptide (VIP).

VIP, a 28-amino acid peptide, was first identified in the porcine intestine in 1970 [15]. Since then, its presence has been described in reproductive, digestive, respiratory, immune, and circulatory systems as well as in the neurons of the central and peripheral nervous systems [16,17,18]. It is worth emphasizing that in the GI tract, VIP acts as both a neurotransmitter and a gastrointestinal hormone, belonging to the group of the so-called brain-gut hormones [16]. VIP is an inhibitory neurotransmitter well-known for its vasodilator effect and its inhibition of smooth muscle contractility [19]. In addition, VIP has a beneficial effect on the integrity of the gastrointestinal mucosa, protects the gastric and duodenal mucosa, and promotes ulcer healing [20]. Furthermore, it is noteworthy that VIP is an important factor involved in the control of various pathological processes. Previous reports have confirmed its participation in defense processes in the central and peripheral nervous systems, with an emphasis on its neuroprotective properties [13,21]. Moreover, VIP exhibits an anti-inflammatory effect, and an increase in VIP expression was noted in the course of numerous inflammatory processes, as well as in the neoplastic changes in the intestines and during the exposure of the body to toxins [11,13,14,22,23]. Bearing in mind the abovementioned properties of VIP and its widespread occurrence in the ENS structures in the small intestine (which is the main site of absorption of the acrylamide contained in food products), we may suspect that it may be involved in defending ENS neurons from the damaging effects of acrylamide. Thus, the present study aims to establish the effect of ACM supplementation in a tolerable daily intake (TDI) dose and a dose ten times higher than TDI on the population of VIP-like immunoreactive (VIP-LI) neurons in the porcine small intestine. Additionally, the degree of the co-localization of VIP with other neuroactive substances known for their neuroprotective properties (neuronal nitric oxide synthase (nNOS), substance P (SP) and cocaine- and amphetamine-regulated transcript peptide (CART)) is studied. This study is conducted using a pig model due its similar anatomical structure and life processes, and the fact that the pig is an omnivorous animal used extensively in the study of gastrointestinal diseases [24].

## 2. Results

### 2.1. The Number of VIP-Positive ENS Neurons

**Figure 1:**
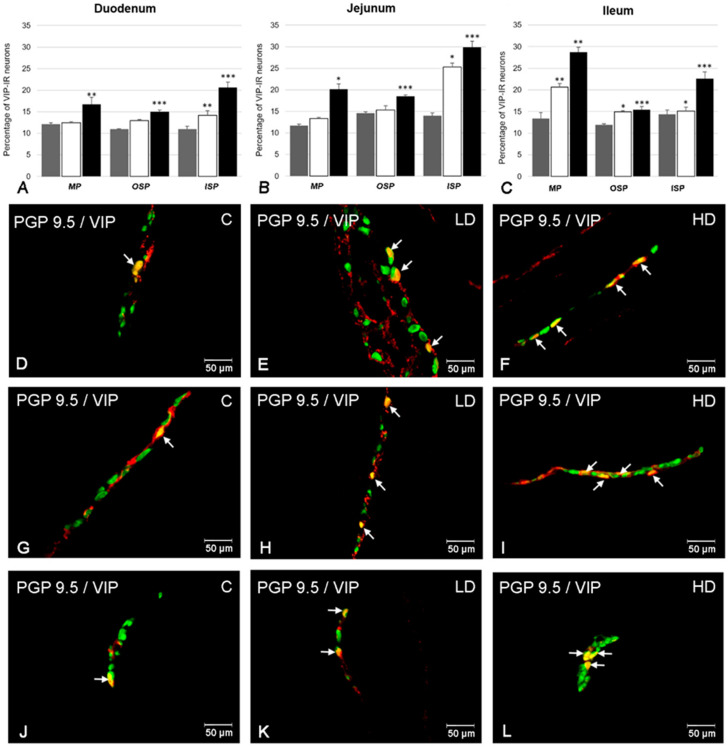
The number of vasoactive intestinal peptide-like immunoreactive (VIP-LI) neurons in the myenteric plexus (MP), outer submucous plexus (OSP), and inner submucous plexus (ISP) in each part of the porcine small intestine (duodenum, jejunum, and ileum) in all experimental groups (**A**–**C**) and the most representative images showing neurons immunoreactive to protein gene-product 9.5 (PGP 9.5)—used as a pan-neuronal marker and vasoactive intestinal peptide (VIP) in animals from control (**D**,**G**,**J**), low-dose (LD) (**E**,**H**,**K**) and high-dose (HD) (**F**,**I**,**L**) group. (**A**) Mean (±SEM) percentage of VIP-LI neurons in the duodenum in animals from control (grey bar), LD (white bar) and HD (black bar) group; (**B**) Mean (±SEM) percentage of VIP-LI neurons in the jejunum in animals from control (grey bar), LD (white bar), and HD (black bar) group; (**C**) Mean (±SEM) percentage of VIP-LI neurons in the ileum in animals from control (grey bar), LD (white bar), and HD (black bar) group; (**D**) VIP-LI neurons in the MP of the ileum in animals from control group; (**E**) VIP-LI neurons in the MP of the ileum in animals from LD group; (**F**) VIP-LI neurons in the MP of the ileum in animals from HD group; (**G**) VIP-LI neurons in the OSP of the ileum in animals from control group; (**H**) VIP-LI neurons in the OSP of the ileum in animals from LD group; (**I**) VIP-LI neurons in the OSP of the ileum in animals from HD group; (**J**) VIP-LI neurons in the ISP of the duodenum in animals from control group; (**K**) VIP-LI neurons in the ISP of the duodenum in animals from LD group; (**L**) VIP-LI neurons in the ISP of the duodenum in animals from HD group. All images were created by digital superimposition of two color channels (green for PGP 9.5 and red for VIP). Intramural neurons positive to VIP are indicated with arrows. Significant differences were assessed with one-way analysis of variance (ANOVA) with Dunnett’s test (* *p* < 0.05, ** *p* < 0.01, *** *p* < 0.001).

The presence of neurons showing immunoreactivity to VIP was confirmed in all intramural plexuses studied (the MP, OSP, and ISP) in each part of the porcine small intestine (duodenum, jejunum, and ileum) (Figure 1). In the MP, the population of VIP-positive neurons accounted for 12.14 ± 0.31% of all PGP 9.5 positive neurons in the duodenum (Figure 1A). A similar number of VIP-LI cell bodies were observed in the jejunum (11.70 ± 0.33%) (Figure 1B) and ileum (13.40 ± 1.21%) (Figure 1C,D). In the OSP, the most numerous group of VIP-LI neurons was observed in the jejunum (14.54 ± 0.33%) (Figure 1B) and slightly fewer in the ileum (11.90 ± 0.29%) (Figure 1C,G) and duodenum (11.00 ± 0.08%) (Figure 1A), whereas in the ISP, higher numbers of VIP-LI cell bodies were noted in the ileum (14.38 ± 0.98%) (Figure 1C) and jejunum (14.02 ± 0.64%) (Figure 1B). The least numerous population of VIP-positive neurons was found in the duodenum (10.96 ± 0.65%) (Figure 1A,J).

Acrylamide supplementation affected the immunohistochemical characteristics of ENS neurons in the porcine small intestine. An increase in the number of neurons showing immunoreactivity towards VIP was noted in all studied fragments (Figure 1). The most remarkable changes were noted in the ISP, in which a statistically important increase was observed in both experimental groups (LD and HD) in all parts of the intestine (duodenum: from 10.96 ± 0.65% in the C group to 14.21 ± 1.03% in the LD group and to 20.65 ± 1.23% in the HD group (Figure 1A,J–L); jejunum: from 14.02 ± 0.64% to 25.27 ± 0.94% and to 29.92 ± 1.32% (Figure 1B); ileum: from 14.38 ± 0.98% to 15.09 ± 0.91% and 22.62 ± 1.52% (Figure 1C), respectively). In the OSP, a statistically important increase was noted in animals receiving low and high doses of acrylamide only in the ileum (from 11.90 ± 0.29% to 14.93 ± 0.26% and to 15.45 ± 0.69%) (Figure 1C,G–I), whereas in the duodenum and jejunum, only in the HD group was the increase statistically significant (from 11.00 ± 0.08% to 14.96 ± 0.45% (Figure 1A) and from 14.54 ± 0.33% to 18.47 ± 0.37% (Figure 1B), respectively). Similarly, in the MP, increased numbers of VIP-LI cell bodies were noted in both LD and HD groups only in the ileum (from 13.40 ± 1.38% in group C to 20.68 ± 0.81% in the LD group and to 28.7 ± 1.21% in the HD group) (Figure 1C–F). In the duodenum and jejunum in the HD group, the increase was also significant (from 12.14 ± 0.31% to 16.71 ± 1.63% (Figure 1A) and from 11.70 ± 0.33% to 20.14 ± 1.27% (Figure 1B).

### 2.2. The Co-Localization of VIP with nNOS

**Figure 2:**
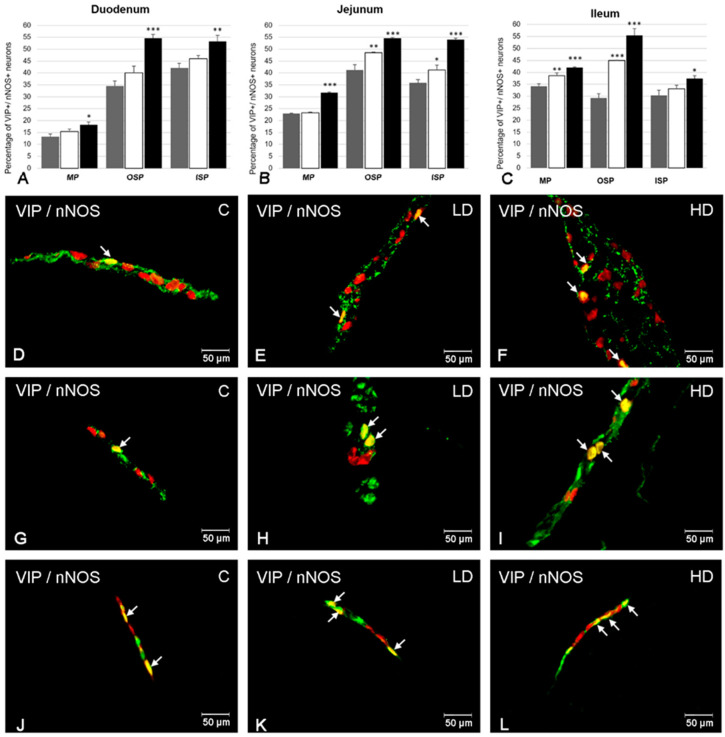
The ratio of co-localization of VIP with nNOS in intramural neurons in each part of the porcine small intestine (duodenum, jejunum and ileum) in control animals (grey bars), after low (white bars) and high doses (black bars) of acrylamide supplementation (**A**–**C**) and the most representative images showing VIP-LI neurons simultaneously immunoreactive to nNOS in animals from control (**D**,**G**,**J**), LD (**E**,**H**,**K**) and HD (**F**,**I**,**L**) group. (**A**) Mean (±SEM) percentage of VIP+/nNOS+ neurons in the myenteric plexus (MP), outer submucous plexus (OSP), and inner submucous plexus (ISP) within the porcine duodenum; (**B**) Mean (±SEM) percentage of VIP+/nNOS+ neurons in the myenteric plexus (MP), outer submucous plexus (OSP), and inner submucous plexus (ISP) within the porcine jejunum; (**C**) Mean (±SEM) percentage of VIP+/nNOS+ neurons in the myenteric plexus (MP), outer submucous plexus (OSP), and inner submucous plexus (ISP) within the porcine ileum; (**D**) VIP+/nNOS+ neurons in the myenteric plexus of the porcine ileum in animals from control group; (**E**) VIP+/nNOS+ neurons in the myenteric plexus (MP) of the porcine ileum in animals from LD group; (**F**) VIP+/nNOS+ neurons in the myenteric plexus (MP) of the porcine ileum in animals from HD group; (**G**) VIP+/nNOS+ neurons in the outer submucous plexus (OSP) of the porcine jejunum in animals from control group; (**H**) VIP+/nNOS+ neurons in the outer submucous plexus (OSP) of the porcine jejunum in animals from LD group; (**I**) VIP+/nNOS+ neurons in the outer submucous plexus (OSP) of the porcine jejunum in animals from HD group; (**J**) VIP+/nNOS+ neurons in the inner submucous plexus (ISP) of the porcine jejunum in animals from control group; (**K**) VIP+/nNOS+ neurons in the inner submucous plexus (ISP) of the porcine jejunum in animals from LD group; (**L**) VIP+/nNOS+ neurons in the inner submucous plexus (ISP) of the porcine jejunum in animals from control group. All images were created by digital superimposition of two color channels (green for VIP and red for nNOS). Intramural neurons immunopositive to VIP and nNOS are indicated with arrows. Significant differences were assessed with one-way analysis of variance (ANOVA) with Dunnett’s test (* *p* < 0.05, ** *p* < 0.01, *** *p* < 0.001).

nNOS immunoreactivity was noted in VIP-positive ENS neurons in each kind of enteric plexus and all parts of the studied intestine (Figure 2). A higher number of VIP+/nNOS+ neurons in the MP were found in the ileum (34.20 ± 1.00%) (Figure 2C,D). A smaller population of neurons simultaneously containing VIP and nNOS were observed in the jejunum (22.91 ± 0.25%) (Figure 2B) and the duodenum (13.11 ± 1.31%) (Figure 2A). In the OSP, the most numerous group of VIP-LI neurons showing the presence of nNOS was detected in the jejunum (41.20 ± 2.19%) (Figure 2B,G), while slightly less were observed in the duodenum (34.38 ± 2.31%) (Figure 2A) and the least were in the ileum (29.30 ± 1.77%) (Figure 2C). In turn, in the ISP, in the duodenum 41.98 ± 2.12% of VIP-LI neurons were nNOS-positive (Figure 2A); in the jejunum the levels were 35.92 ± 1.35% (Figure 2B,J), and in the ileum the levels were 30.38 ± 2.21% (Figure 2C).

Acrylamide caused significant changes in the number of VIP-positive neurons that are simultaneously immunoreactive to nNOS (Figure 2). In the MP, the most remarkable changes in the percentage of VIP+/nNOS+ neurons were observed in the ileum, in which both doses of acrylamide evoked a significant increase (to 38.68 ± 1.13% in the LD group and to 42.07 ± 0.25% in the HD group) (Figure 2C–F). However, in the duodenum and jejunum, important changes were observed only in the HD group (an increase to 18.28 ± 1.12% (Figure 2A) and to 31.70 ± 0.33% (Figure 2B), respectively). In the OSP, both doses of acrylamide changed the degree of co-localization of VIP and nNOS in the ileum (to 44.93 ± 0.26% in the LD group and to 55.45 ± 2.8% in the HD group) (Figure 2C) as well as in the jejunum (to 48.47 ± 0.37% in the LD group and to 54.54 ± 0.33% in the HD group) (Figure 2B,G–I), while in the duodenum only a high dose of acrylamide evoked a significant increase in the number of VIP+/nNOS+ cell bodies (to 54.62 ± 1.53%) (Figure 2A). In turn, in the ISP, only in the jejunum in both experimental groups was an increased population of VIP-LI neurons simultaneously containing nNOS noted (to 41.27 ± 2.03% in the LD group and to 54.02 ± 0.64% in the HD group) (Figure 2B,J-L). However, only supplementation of a high dose of acrylamide changed the ratio of VIP+/nNOS+ neurons in the duodenum and ileum (an increase to 53.27 ± 2.45% and 37.42 ± 1.21%, respectively) (Figure 2A,C).

### 2.3. The Co-Localization of VIP with SP

**Figure 3:**
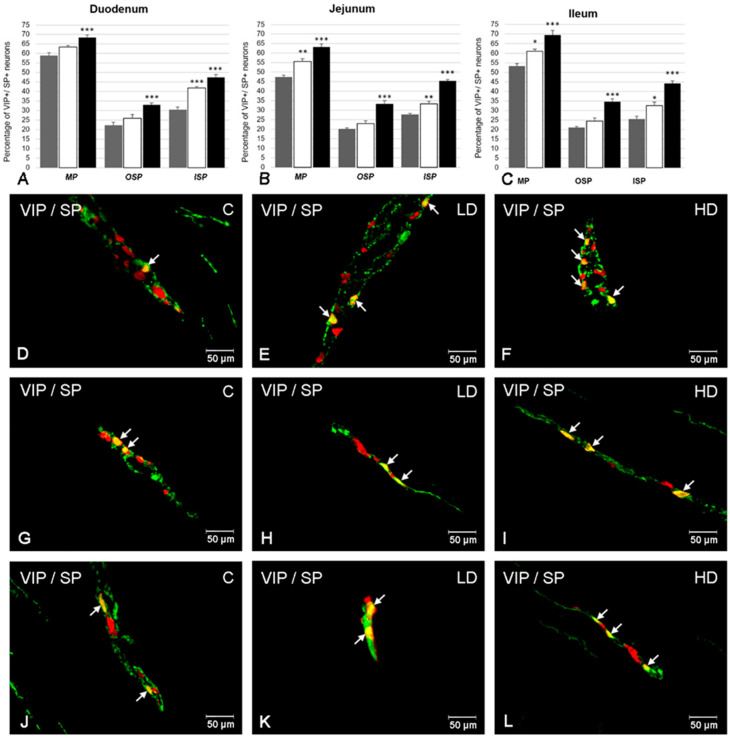
The ratio of co-localization of VIP with SP in intramural neurons in each part of the porcine small intestine (duodenum, jejunum, and ileum) in control animals (grey bars), after low (white bars) and high doses (black bars) of acrylamide supplementation (**A**–**C**) and the most representative images showing VIP-LI neurons simultaneously immunoreactive to substance P (SP) in animals from control (**D**,**G**,**J**), LD (**E**,**H**,**K**), HD (**F**,**I**,**L**) group. (**A**) Mean (±SEM) percentage of VIP+/SP+ neurons in the myenteric plexus (MP), outer submucous plexus (OSP), and inner submucous plexus (ISP) within the porcine duodenum; (**B**) Mean (±SEM) percentage of VIP+/SP+ neurons in the myenteric plexus (MP), outer submucous plexus (OSP), and inner submucous plexus (ISP) within the porcine jejunum; (**C**) Mean (±SEM) percentage of VIP+/SP+ neurons in the myenteric plexus (MP), outer submucous plexus (OSP), and inner submucous plexus (ISP) within the porcine ileum; (**D**) VIP+/SP+ neurons in the myenteric plexus of the porcine jejunum in animals from control group; (**E**) VIP+/SP+ neurons in the myenteric plexus of the porcine jejunum in animals from LD group; (**F**) VIP+/SP+ neurons in the myenteric plexus of the porcine jejunum in animals from HD group; (**G**) VIP+/SP+ neurons in the outer submucous plexus (OSP) of the porcine duodenum in animals from control group; (**H**) VIP+/SP+ neurons in the outer submucous plexus (OSP) of the porcine duodenum in animals from LD group; (**I**) VIP+/SP+ neurons in the outer submucous plexus (OSP) of the porcine duodenum in animals from HD group; (**J**) VIP+/SP+ neurons in the inner submucous plexus (ISP) of the porcine duodenum in animals from control group; (**K**) VIP+/SP+ neurons in the inner submucous plexus (ISP) of the porcine duodenum in animals from LD group; (**L**) VIP+/SP+ neurons in the inner submucous plexus (ISP) of the porcine duodenum in animals from HD group. All images were created by digital superimposition of two color channels (green for VIP and red for SP). Intramural neurons immunopositive to VIP and SP are indicated with arrows. Significant differences were assessed with one-way analysis of variance (ANOVA) with Dunnett’s test (* *p* < 0.05, ** *p* < 0.01, *** *p* < 0.001).

In the control group, a higher degree of co-localization of VIP with SP was noted in the MP in the duodenum (58.74 ± 1.69%) (Figure 3A) and slightly less in the ileum (53.33 ± 1.17) (Figure 3C) and the jejunum (47.42 ± 0.88%) (Figure 3B,D). Similarly, in the OSP, a higher population of VIP+/SP+ neurons were observed in the duodenum (22.27 ± 1.66%) (Figure 3A,G), than in the ileum (21.1 ± 0.6%) (Figure 3C) or the jejunum (20.24 ± 0.57%) (Figure 3B). In the ISP, the co-localization of VIP with SP was also the highest in the duodenum (30.46 ± 1.37%) (Figure 3A,J), followed by the jejunum (27.73 ± 0.66%) (Figure 3B) and the ileum (25.58 ± 1.48%) (Figure 3C).

Supplementation of both low and high doses of acrylamide evoked changes in the proportion of co-localization of VIP with SP in all kinds of intestinal plexuses examined (Figure 3). Both the low and high doses of acrylamide increased the population of VIP+/SP+ neurons in the ISP in all sections of the intestine studied. A higher increase was noted in the duodenum (to 41.94 ± 0.77% in the LD group and to 47.53 ± 1.38% in the HD group) (Figure 3A,J–L), slightly smaller in the jejunum (to 33.33 ± 1.28% in the LD group and to 45.40 ± 0.74% in the HD group) (Figure 3B) and the smallest in the ileum (to 32.62 ± 1.86% in the LD group and to 44.22 ± 1.31% in the HD group) (Figure 3C). In the MP, the changes were significant in both experimental groups in the jejunum (an increase to 55.57 ± 1.55% in LD group and to 63.25 ± 1.64% in the HD group) (Figure 3B,D–F) and the ileum (an increase to 61.02 ± 1.05% in the LD group and to 69.59 ± 2.30% in the HD group) (Figure 3C). However, in the duodenum only in the HD group was the increase remarkable (to 68.35 ± 1.40%) (Figure 3A). In turn, in the OSP, in all fragments of the intestine under investigation, only in the HD group was an increased number of VIP-LI neurons simultaneously immunopositive to SP observed (an increase to 33.06 ± 1.05% in the duodenum (Figure 3A,G–I), to 33.34 ± 1.66% in the jejunum (Figure 3B) and to 34.6 ± 1.4% in the ileum (Figure 3C), respectively).

### 2.4. The Co-Localization of VIP with CART

**Figure 4:**
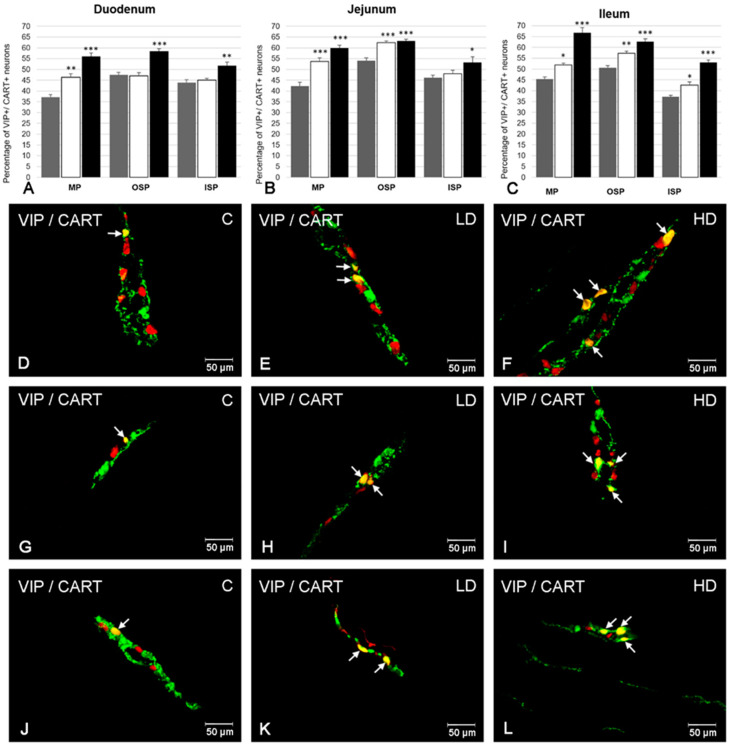
The ratio of co-localization of VIP with CART in intramural neurons in each part of the porcine small intestine (duodenum, jejunum, and ileum) in control animals (grey bars), after low (white bars) and high dose (black bars) of acrylamide supplementation (**A**–**C**) and the most representative images showing VIP-LI neurons simultaneously immunoreactive to CART in animals from control (**D**,**G**,**J**), LD (**E**,**H**,**K**), HD (**F**,**I**,**L**) group. (**A**) Mean (±SEM) percentage of VIP+/CART+ neurons in the myenteric plexus (MP), outer submucous plexus (OSP), and inner submucous plexus (ISP) within the porcine duodenum; (**B**) Mean (±SEM) percentage of VIP+/CART+ neurons in the myenteric plexus (MP), outer submucous plexus (OSP), and inner submucous plexus (ISP) within the porcine jejunum; (**C**) Mean (±SEM) percentage of VIP+/CART+ neurons in the myenteric plexus (MP), outer submucous plexus (OSP), and inner submucous plexus (ISP) within the porcine ileum; (**D**) VIP+/CART+ neurons in the myenteric plexus of the porcine duodenum in animals from control group; (**E**) VIP+/CART+ neurons in the myenteric plexus of the porcine duodenum in animals from LD group; (**F**) VIP+/CART+ neurons in the myenteric plexus of the porcine duodenum in animals from HD group; (**G**) VIP+/CART+ neurons in the outer submucous plexus (OSP) of the porcine jejunum in animals from control group; (**H**) VIP+/CART+ neurons in the outer submucous plexus (OSP) of the porcine jejunum in animals from LD group; (**I**) VIP+/CART+ neurons in the outer submucous plexus (OSP) of the porcine jejunum in animals from HD group; (**J**) VIP+/CART+ neurons in the inner submucous plexus (ISP) of the porcine ileum in animals from control group; (**K**) VIP+/CART+ neurons in the inner submucous plexus (ISP) of the porcine ileum in animals from LD group; (**L**) VIP+/CART+ neurons in the inner submucous plexus (ISP) of the porcine ileum in animals from HD group. All images were created by digital superimposition of two color channels (green for VIP and red for CART). Intramural neurons immunopositive to VIP and CART are indicated with arrows. Significant differences were assessed with one-way analysis of variance (ANOVA) with Dunnett’s test (* *p* < 0.05, ** *p* < 0.01, *** *p* < 0.001).

Another substance co-localizing with VIP in intramural neurons was CART (Figure 4). In the MP, the percentage of neurons positive to both neuroactive substances was estimated at 37.25 ± 1.25% in the duodenum (Figure 4A,D), 42.32 ± 1.68% in the jejunum (Figure 4B) and 45.44 ± 0.97% in the ileum (Figure 4C), respectively. In the OSP, the number of VIP-LI neurons that simultaneously expressed CART was similar in the jejunum (53.97 ± 1.41%) (Figure 4B,G) and ileum (50.59 ± 0.92%) (Figure 4C). Slightly fewer VIP+/CART+ neurons were noted in the duodenum (47.45 ± 1.15%) (Figure 4A). In turn, in the ISP, a higher degree of co-localization of VIP with CART was observed in the jejunum (46.18 ± 1.09%) (Figure 4B) and duodenum (43.81 ± 1.36%) (Figure 4A), while in the ileum this value amounted to 37.20 ± 0.66% (Figure 4C,J).

In the experimental groups, the number of VIP-LI neurons simultaneously immunopositive to CART increased in all segments of the small intestine and types of enteric plexus examined (Figure 4). The most visible changes were noted in the MP in both experimental groups and all segments of the small intestine, reaching the following values: in the duodenum, 46.41 ± 1.64% in the LD group and 55.97 ± 1.61% in the HD group (Figure 4A,D–F); in the jejunum, 53.70 ± 1.68% in the LD group and 59.93 ± 1.35% in the HD group (Figure 4B); in the ileum, 51.84 ± 0.92% in the LD group and 66.80 ± 2.30% in the HD group (Figure 4C). In turn, in the OSP both doses of acrylamide led to an increase in the percentage of VIP+/CART+ cell bodies in the jejunum (to 62.30 ± 0.87% in the LD group and to 63.19 ± 0.75% in the HD group) (Figure 4B,G–I) and in the ileum (to 57.23 ± 1.09% in the LD group and to 62.60 ±1.41% in the HD group) (Figure 4C). In the duodenum, only in the HD group an increase was noted (to 58.46 ± 1.18%) (Figure 4A). In turn, in the ISP, only in the ileum was the increase statistically important after supplementation of both acrylamide doses (to 42.56 ± 1.52% in the LD group and to 53.10 ± 1.16% in the HD group) (Figure 4C,J–L), while in the duodenum and jejunum only in the HD group were an elevated number of VIP+/CART+ neurons noted (to 51.74 ± 1.72% (Figure 4A) and to 53.25 ± 2.58 (Figure 4B), respectively).

## 3. Discussion

During this experiment, the presence of VIP was demonstrated in the ENS neurons in the porcine small intestine. In the physiological state, the number of VIP-LI neurons was similar in each kind of enteric plexus studied. This is comparable to previous studies conducted on the gut in the pig [13,14,25]. Moreover, the distribution of VIP in the structures of the ENS in the GI tract has been described in many other species of animals as well as in humans [26,27,28]. This is consistent with the numerous physiological functions of VIP in the digestive tract reported in the literature. First of all, VIP as an inhibitory neuropeptide inhibits gastric and intestinal motility. As the motor function is mainly regulated by the myenteric plexus, the VIP synthesized in the myenteric plexus is, with high probability, responsible for the inhibitory effect on the motor function. By contrast, VIP present in submucous neurons regulates the secretory function, such as ion secretion and fluid flow in the pancreas and intestine, and inhibits the secretion of digestive juices [19,29]. Additionally, VIP participates in the regeneration and control tight junction barrier function of the intestinal epithelium [30]. The multiple physiological functions of VIP are mediated via one of G-protein coupled receptors: VPAC1 (VIP/PACAP receptor, subtype 1) and VPAC2 (VIP/PACAP receptor) receptors binding VIP and PACAP, causing an increase in cyclic AMP and activating the protein kinase A pathway [31]. It was confirmed that most of the VIP functions in the intestine are performed through the VPAC1 receptor [32]. Furthermore, in the control group, VIP-positive ENS neurons simultaneously expressed other studied neuroactive substances. Previous studies showed that neurotransmitters synthetized in the same neurons possess a similar or auxiliary function [33]. The coexistence of nNOS, SP, and CART with VIP in ENS neurons in the small intestine suggests their complicity in the regulation of biological functions of this part of the GI tract in the pig.

The current study provides, for the first time, comprehensive data describing the effect of acrylamide supplementation on the population of VIP-LI neurons in the porcine small intestine. Both acrylamide doses (TDI and ten times higher) evoked changes in the expression of VIP in enteric neurons in each type of enteric plexus under investigation (i.e., MP, OSP, ISP). It is worth noting that inflammatory processes, neuronal damage, and many other harmful factors that the digestive tract is exposed to evoke functional changes in ENS neurons. These adaptive changes, including neuronal hyperplasia, degeneration of nerve endings, down or upregulation of neurotransmitter synthesis, and expression of their receptors, are an important line of defense and are known as neuronal plasticity [34]. The numerous studies conducted so far show that ACM has an adverse effect on living organisms, namely in demonstrating neurotoxic effects, reproductive disorders, and carcinogenicity [1,4,5,9]. Among them, neurotoxicity is of particular interest to researchers because this toxic effect has been described in both laboratory animals and humans (occupational exposure). The main clinical symptoms of ACM neurotoxicity include weakness, tingling and numbness in the extremities, convulsions, and ataxia [7]. It has been previously established that ACM led to the apoptosis of cerebellar Purkinje cells and the degeneration of axons and nerve terminals in the central and peripheral nervous systems [7,9]. Subsequent studies showed that ACM disrupted the neurotransmission by the inhibition of neurotransmitter release from the presynaptic membrane, i.e., reuptake and vesicular storage mainly via binding to nucleophilic cysteine sites on proteins [7,35]. Moreover, ACM inhibits Na+/K+-ATPase action [36] and fast axonal transport [37] and interferes with the work of enzymes involved in neuronal energy production [38]. Considering the known toxic effects of acrylamide on neurons, we can speculate that the increased number of VIP-LI neurons observed in the present study may have resulted from an inhibition of axonal transport induced by acrylamide. It is also highly probable that alteration in VIP immunoreactivity is a result of the increased synthesis of VIP in neuronal cells at various stages of this process (i.e., transcription, translation, or changes in the activity of enzymes involved in the synthesis) as a response on direct irritant effect of acrylamide on ENS neurons. The neuroprotective effect of VIP has been studied for many years in different experimental models. VIP exerts a potent neuroprotective effect through the activation of different signaling pathways and transcriptional-genetic activity. For instance, VIP decreases Aβ accumulation and atrophy in the hippocampus and cortex in mice models of Alzheimer’s disease [39], and it increases the spine density and prevents dopaminergic cell loss in rat models of Parkinson’s disease [40]. Additionally, the survival effect of VIP on adult rat myenteric neurons in a culture was also confirmed [41]. The obtained results are comparable to previous results concerning the alteration of VIP immunoreactivity in enteric structures following pathophysiological situations through the digestive tract. Altered VIP levels in the ENS structure were found during nerve damage [13,22], diabetes [25], gastric ulcer [42], and bisphenol A intoxication [14].

Furthermore, numerous studies indicate that ACM, by the accumulation of excessive reactive oxygen species (ROS), causes oxidative stress and leads to damage in different organs of the body, including the brain, liver, and kidneys [43]. Notably, oxidative stress and enhanced lipid peroxidation are one of the main mechanisms of ACR-induced neurotoxicity [44]. The results obtained in the present study may be a response of ENS neurons to oxidative stress. Previous studies have indicated the potential role of VIP as a therapeutic agent that reduces oxidative stress and prevents cell damage caused by ROS [40,45].

Due to the anti-inflammatory properties of VIP, it can also be speculated that the increase in VIP-immunoreactivity in the ENS neurons is a response to local inflammation induced by acrylamide supplementation. The authors’ earlier studies showed that acrylamide intoxication is accompanied by an increase in the synthesis of proinflammatory cytokines in the ileum wall [46]. Likewise, in human blood, an elevated concentration of high-sensitivity interleukin-6, high-sensitivity C-reactive protein, and gamma-glutamyl transferase concentrations was observed during the chronic intake of potato chips rich in acrylamide [47]. Earlier studies confirmed the participation of VIP in cross-talk between the ENS and enteric immune system. VIP effects were found on immune and inflammatory responses by promoting T helper cell differentiation, stimulating regulatory T cell synthesis, and downregulating macrophage action [34]. VIP was also shown to inhibit proinflammatory cytokine release (such as IL-6, TNF-α, IL-12) and increase IL-10 synthesis [34,48]. Additionally, VIP exerts its anti-inflammatory effect by regulation of the epithelial barrier function, mainly by increasing the secretion of ions and fluids in the intestines and control of epithelial tight junction proteins synthesis [34,49]. There is also evidence that VIP acts directly on microglial cells as microglia-deactivating factors and, in this way, protect neurons against neuroinflammation [50]. Likewise, upregulated expression of VIP in the ENS structures was noted during inflammatory states in the GI tract, such as Helicobacter pylori infection [51], Crohn’s disease [52], and ulcerative colitis [53].

In the current experiment, acrylamide-inducted alterations in the degree of the co-localization of VIP with nNOS, SP, and CART in the intramural neurons through the porcine small intestine was also demonstrated. Acrylamide supplementation increased the number of VIP-LI neurons simultaneously containing nNOS in all intramural plexuses examined. Changes were visible in both experimental groups and depended on the kind of plexus and the part of the intestine under investigation. Several lines of evidence show that NO possesses neuroprotective properties and also has a regulatory role in many inflammatory processes [33,54]. To date, an increased number of nNOS-LI enteric neurons were noted in inflammatory bowel disease [55] after denervation [56] or bisphenol A administration [57]. Similar to VIP, nNOS has a beneficial effect on the survival rate of cultured rat colonic submucous neurons [58]. However, some pathological conditions in the gut, such as diabetes or Crohn’s disease, may lead to a reduction in the population of nitrergic neurons in the gut wall [59]. It is highly probable that the role of nNOS in the control of pathological conditions in the GI tract depends on the type of harmful factors and type of inflammation as well as the section of the GI tract studied.

Another substance co-localized with VIP in the ENS neurons in the porcine small intestine was SP. In this case, both doses of acrylamide also led to an increase in the population of VIP+/SP+ neurons in all studied enteric plexuses. The changes were the most significant in the ISP and MP. However, in the OSP, only a high dose caused significant changes. Alterations in the SP level in the nervous structures located in the intestinal wall have been described in numerous pathological states, such as inflammatory bowel disease [60], Bacteroides fragilis [61] and Trypanosoma cruzi infection [62], bisphenol A intoxication [14], and neoplastic diseases [63]. Previous studies have indicated the possible role of SP in the control of inflammatory conditions. SP stimulates the synthesis of TNF-α, IL-1β, and IL-6 in the human colonic mucosa [64], increases the production of IL-1β and IL-6 mRNA in human glial cells, and co-operates with mast cells in the wall of ileum and ascending colon of irritable bowel syndrome (IBS) patients [65,66]. Therefore, the observed changes in the present study in a population of VIP+/SP+ neurons may be associated with local inflammation caused by acrylamide supplementation. However, since SP is involved in pain transmission, it can also be speculated that the observed changes may be a result of a pain condition accompanying acrylamide intoxication.

Finally, a significant increase in the number of VIP-LI neurons simultaneously containing CART was observed after the administration of both acrylamide doses, and the changes were the most remarkable in the MP. Slightly smaller changes were noted in the submucous plexuses. In recent years, many studies have revealed that CART exerts neuroprotective effects. It was established that CART is engaged in protection against the ischemic cerebral injury by reducing inflammation activation and inhibiting oxidative stress and protecting mitochondrial functions [67]. Changes in CART immunoreactivity in both nerve fibers, as well as neuron cell bodies in the GI tract wall, were also described following numerous pathological conditions. An upregulated level of CART accompanying proliferative enteropathy of the descending colon [68] and axotomy [69] in pigs as well as hypertension in rats [70] were noted. Additionally, Ekblad [71] reported that CART enhances neuronal survival of culturing myenteric neurons in rats. Although the mechanism of CART neuroprotective action has not been fully elucidated, the research carried out to date and the results of this experiment suggest that it plays a neuroprotective and neurotrophic role in the intestines. Based on the well-known anti-inflammatory and neuroprotective activities of VIP, it can be concluded that they are involved in the protection of ENS neurons against the harmful effects of acrylamide in the porcine small intestine and cooperate in this process with nNOS, SP, and CART. The obtained results here are the basis for further pharmacological and clinical study. In the future, agonists of VPAC receptors may be potentially useful agents in managing the toxic effects evoked by acrylamide supplementation. However, further research is needed to better understand the function and the exact mechanism of action of these substances in the course of acrylamide intoxication.

## 4. Material and Methods

In the experiment, 15 Danish landrace gilts purchased from a local farm and of about 15 kg body weight (b.w.) were used. The animals were group-housed in age-appropriate cages, fed commercial feed, and had constant access to water. All experimental procedures carried out on animals were approved by the Local Commission for Animal Experiments in Olsztyn (decision no. 11/2017, 28 February 2017). After a 7-day acclimatization period, the animals were assigned to one of three experimental groups: (a) the control (C group), i.e., gilts receiving empty gelatin capsules; (b) a low-dose group (LD group, *n* = 5), gilts receiving a tolerable daily intake (TDI) dose (0.5 µg/kg b.w./day) of acrylamide (>99%; Sigma-Aldrich, Poznań, Poland) in gelatin capsules; and (c) a high-dose group (the HD group, *n* = 5), i.e., gilts receiving a high dose of acrylamide (ten times higher than TDI, i.e., 5 µg/kg b.w./day) in gelatin capsules. The capsules were administered in the same manner to all animals for a period of 28 days with their morning feeding. After this time, the gilts of all groups were euthanized with sodium pentobarbital (Morbital, Biowet Puławy, Puławy, Poland), and fragments of the small intestine (duodenum, jejunum, and ileum) were collected for further examination. Tissues were fixed by immersion in a 4% buffered solution of paraformaldehyde (pH 7.4) for one hour and transferred to a phosphate buffer solution (PBS, pH 7.4) for 72 h (a buffer was exchanged 3 times, every 24 h). The tissues were then placed into an 18% buffered sucrose solution for two weeks. After this period, 14 µm thick frozen sections were prepared and subjected to the procedure of double immunofluorescence staining as described previously by Palus et al. [72].

Briefly, sections were dried at room temperature for 45 min, then washed in a buffer solution (PBS, 3 times, 10 min), blocked in a blocking mixture (10% horse serum, 0.1% bovine serum albumin in 0.1 M PBS, 1% Triton X-100, 0.05% thimerosal, and 0.01% sodium aside) for 1 h, rinsed again in the PBS (3 times, 10 min), and then placed into the primary antibody mixture consisting of a protein gene-product 9.5 (PGP 9.5; mouse, cat. No. 7863-2004, Bio-Rad, Hercules, CA, USA, working dilution 1:1000, used here as a pan-neuronal marker) and vasoactive intestinal peptide (VIP; rabbit, cat. No. 11428, Cappel, Aurora, OH, USA, working dilution 1:3000), as well as VIP (mouse, cat. No. 9535–0504, Biogenesis Inc., Poole, UK, working dilution 1: 2000), neuronal nitric oxide synthase (nNOS; rabbit, cat. No. AB5380, Sigma-Aldrich, Saint Louis, MO, USA, working dilution 1:2000), substance P (SP, rat monoclonal, AbD Serotec, Raleigh, NC, USA; Cat. # 8450-0505; working dilution 1:150), cocaine- and amphetamine-regulated transcript peptide (CART; rabbit, cat. No. H-003-61, Phoenix Pharmaceuticals, Burlingame, CA, USA, working dilution 1:8000), and incubated overnight in a humid chamber.

On the second day, the sections were washed in the PBS (three times, 10 min) and incubated with a mixture of secondary antibodies (Alexa Fluor 488 (donkey anti-mouse IgG, cat. No. A21202, Invitrogen, Carlsbad, CA, USA, working dilution 1:1000)), Alexa Fluor 546 (goat anti-rabbit IgG, cat. No. A11010, Invitrogen, Carlsbad, CA, USA, working dilution 1:1000), and Alexa Fluor 546 (donkey anti-rat, cat. No. A21208, Invitrogen, Carlsbad, CA, USA, working dilution 1:1000) at room temperature for 1 h. After another wash in the PBS, the sections were coverslipped using a mixture of glycerol with carbonate buffer (pH = 8.4). To assess the specificity of the method, standard controls were used, including replacement, omission, and preabsorption tests.

The stained sections were analyzed under an epifluorescence microscope and then photographed using a digital camera connected to a computer with Olympus Cell F image analysis software (Olympus, Tokyo, Japan). The number of ENS neurons showing immunoreactivity against VIP in particular plexus types (i.e., MP, ISP, OSP) in all three sections of the small intestine (duodenum, jejunum, ileum) was determined as the percentage of PGP 9.5 immunoreactive neurons (pan-neuronal marker). No less than 500 PGP 9.5 neurons in each of the analyzed plexuses were used for counting in sections separated at least 200 µm from each other. The degree of VIP co-localization with nNOS, SP, and CART was determined by counting at least 100 VIP-LI neurons in each plexus type (MP, ISP, OSP) for all antisera combinations. In this analysis, VIP-LI neurons were considered as 100%. Data were pooled and expressed as a mean ± standard error of mean (SEM). Statistical analysis were performed with Statistica 12 software (StatSoft Inc., Tulsa, OK, USA) using a one-way analysis of variance (ANOVA) with Dunnett’s test (* *p* < 0.05, ** *p* < 0.01, *** *p* < 0.001).

## 5. Conclusions

In conclusion, acrylamide administration increased the population of VIP-LI intramural neurons in the porcine small intestine. Moreover, both doses of acrylamide led to changes in the degree of co-localization of VIP with nNOS, SP, and CART in all types of ENS plexuses and fragments of small intestine studied. The observed changes may be the adaptation of neurons to local inflammation, oxidative stress or the direct toxic effects of acrylamide on intestinal neurons referred to as neuronal plasticity. The low doses of acrylamide used in the experiment (corresponding to the real human exposure to this toxin) induced a significant response of ENS neurons in all sections of the porcine small intestine. Therefore, it should be considered whether the current legal regulations are sufficient to ensure the safety of food products containing acrylamide. These studies may become the starting point for further toxicological and pharmacological studies. In the future, VIP and/or its receptor agonists may potentially act as therapeutic agents to protect ENS neurons from the harmful effects of acrylamide contained in food products.
